# New insights into the genome and transmission of the microsporidian pathogen *Nosema muscidifuracis*

**DOI:** 10.3389/fmicb.2023.1152586

**Published:** 2023-04-13

**Authors:** Xiao Xiong, Christopher J. Geden, Dan T. Bergstralh, Roxie L. White, John H. Werren, Xu Wang

**Affiliations:** ^1^Fundamental Research Center, Shanghai YangZhi Rehabilitation Hospital, Shanghai Sunshine Rehabilitation Center, School of Life Sciences and Technology, Tongji University, Shanghai, China; ^2^Department of Pathobiology, College of Veterinary Medicine, Auburn University, Auburn, AL, United States; ^3^Center for Medical, Agricultural and Veterinary Entomology, USDA Agricultural Research Service, Gainesville, FL, United States; ^4^Department of Biology, University of Rochester, Rochester, NY, United States; ^5^Department of Entomology and Plant Pathology, College of Agriculture, Auburn University, AL, United States; ^6^Alabama Agricultural Experiment Station, Center for Advanced Science, Innovation and Commerce, Auburn, AL, United States; ^7^HudsonAlpha Institute for Biotechnology, Huntsville, AL, United States

**Keywords:** fungal genomics, hymenopteran fungal pathogens, parasitoid wasps, biological control, *Nosema* disease, microsporidiosis

## Abstract

**Introduction:**

*Nosema* is a diverse genus of unicellular microsporidian parasites of insects and other arthropods. *Nosema muscidifuracis* infects parasitoid wasp species of *Muscidifurax zaraptor* and *M. raptor* (Hymenoptera: Pteromalidae), causing ~50% reduction in longevity and ~90% reduction in fecundity.

**Methods and Results:**

Here, we report the first assembly of the *N. muscidifuracis* genome (14,397,169 bp in 28 contigs) of high continuity (contig N50 544.3 Kb) and completeness (BUSCO score 97.0%). A total of 2,782 protein-coding genes were annotated, with 66.2% of the genes having two copies and 24.0% of genes having three copies. These duplicated genes are highly similar, with a sequence identity of 99.3%. The complex pattern suggests extensive gene duplications and rearrangements across the genome. We annotated 57 rDNA loci, which are highly GC-rich (37%) in a GC-poor genome (25% genome average). *Nosema*-specific qPCR primer sets were designed based on 18S rDNA annotation as a diagnostic tool to determine its titer in host samples. We discovered high *Nosema* titers in *Nosema*-cured *M. raptor* and *M. zaraptor* using heat treatment in 2017 and 2019, suggesting that the remedy did not completely eliminate the *Nosema* infection. Cytogenetic analyses revealed heavy infections of *N. muscidifuracis* within the ovaries of *M. raptor* and *M. zaraptor*, consistent with the titer determined by qPCR and suggesting a heritable component of infection and per ovum vertical transmission.

**Discussion:**

The parasitoids-*Nosema* system is laboratory tractable and, therefore, can serve as a model to inform future genome manipulations of *Nosema*-host system for investigations of Nosemosis.

## Introduction

*Nosema* (Microsporidia: Nosematidae) is one of the most diverse genera of microsporidian parasites, and they are widely distributed unicellular parasites of insects and other arthropods (Didier et al., 1998; Becnel and Andreadis, 1999). *Nosema* infection, a disease known as Nosemosis, occurs throughout the world and leads to agricultural economic losses through detrimental effects on pollinators due to *N. apis* infections found in European honey bee *Apis mellifera* and *N. ceranae* infections originally detected in Asian honey bee *Apis cerana* (Fries et al., 1996; Chen et al., 2013; Pelin et al., 2015; Stanimirović et al., 2019). Another *Nosema* species, *N. bombycis*, infects the silk moth *Bombyx mori*, causing a lethal disease called Pébrine, which is a serious threat to silk production (Pan et al., 2013). The consequences of *Nosema* infection were extensively studied due to its economic importance.

*Nosema* parasitism has destructive effects on honey bees, including digestive disorders, poor colony development, and reduction in metabolism and reproduction (Rinderer and Sylvester, 1978; Malone et al., 1995; Higes et al., 2009). *Nosema ceranae* is more prevalent, impairing bee health and declining bee colonies. It damages the physical and immune barriers of honey bees, making them more vulnerable to other pathogenic factors, which is related to colony collapse (Antúnez et al., 2009; Chen et al., 2009; Costa et al., 2011; Dussaubat et al., 2012; Gómez-Moracho et al., 2021). Despite the low prevalence of *N. apis* compared to *N. ceranae* (Cox-Foster et al., 2007), there is no substantial difference in virulence between the two species, and the negative impacts of *N. apis* on the infected colonies cannot be neglected (Forsgren and Fries, 2010; Botías et al., 2013). *Nosema apis* was reported to be associated with reduced lifespan and increased winter mortality in infected bees, and its negative effects on colony strength and productivity have been described in several studies (Fries et al., 1984; Fries, 1988; Anderson and Giacon, 1992; Farrar, 2014). *Nosema bombycis*-infected larvae are inactive and develop slowly, with black spots spreading all over the bodies, eventually leading to death (Pan et al., 2013). Currently, there is no effective treatment for the disease.

The mechanisms of *Nosema* transmission are directly related to the control of Nosemosis. *Nosema bombycis* can be transmitted from the mother host to their eggs vertically and gradually invades the whole body of the larvae, including muscles, intestines, silk glands, and Malpighian tubules. *Nosema* transmission in honey bees is primarily through the mouth to uninfected bees. *Nosema apis* tends to restrict its life cycle to gut epithelial cells after infection, while *N. ceranae* is mainly distributed in the midgut but also spread in other tissues (Chen et al., 2009; Dussaubat et al., 2012). Due to the limitations of conducting *Nosema* infection experiments in bees, another Hymenoptera-*Nosema* model system is needed for research in controlled laboratory settings to determine the infection, distribution, and transmission of *Nosema* and inform the control of Nosemosis.

*Nosema* also infects other beneficial insects, including the parasitoid wasp genus *Muscidifurax* (Hymenoptera: Pteromalidae). The genus *Muscidifurax* is a natural biocontrol agent of the dipteran filth flies with nine identified species, all of which are pupal parasitoid wasps. *Muscidifurax raptor* Girault and Sanders was the first species characterized in 1910 (Girault and Sanders, 1910). In 1970, four sibling species in this genus were described: *M. zaraptor* Kogan and Legner, from the southwestern United States; *M. raptoroides* Kogan and Legner from Central America and Mexico; *M. raptorellus* Kogan and Legner from Uruguay and Chile; and a thelytokous species *M. uniraptor* Kogan and Legner from Puerto Rico (Kogan and Legner, 1970). *Muscidifurax uniraptor* only produces a female offspring, and the parthenogenesis is caused by an intracellular bacterium which is an A-group *Wolbachia w*Uni (Zchori-Fein et al., 2000; Newton et al., 2016). The microsporidium was first found infecting *M. raptor* collected from New York dairy farms in 1990 (Zchori-Fein et al., 1992), and was later described as *N. muscidifuracis* (Becnel and Geden, 1994). Nosemosis significantly affects the development, longevity, and fecundity of *M. raptor*. *Muscidifurax raptor* infected by *N. muscidifuracis* takes longer to complete development, lives about half as long, and produces about 10% as many progeny as uninfected individuals (Geden et al., 1995).

*Nosema* disease reduction methods have been explored to cure the *N. muscidifuracis* infection. Exposure of parasitoid eggs within host pupae at several temperatures (45°C, 47°C, and 50°C) was shown to be effective in managing *Nosema* disease and increasing parasitoid fecundity (Boohene et al., 2003). A 100% cure rate was achieved at 50°C for 45 min with a relative survival of 18%. To completely eliminate the pathogen, heat treatment in combination with the Pasteur method was applied: heat treatment minimizes disease prevalence and ensures an adequate genetic base for healthy parasitoids, and the Pasteur method (based on visual examination for patent infections) isolates uninfected wasps as parents for rearing their progeny (Geden et al., 1995; Steinhaus, 2012). However, the efficacy of this approach depends on the efficiency of visual detection, which may not detect low-level infections.

Understanding the *Nosema* disease transmission mechanism is critical for developing new control methods, but the transmission of *Nosema* in parasitoid wasps is not fully understood yet. The following is known about transmission patterns: (1) maternal transmission is highly efficient; (2) adults can acquire infection by feeding on spore suspensions or infected parasitoid immatures within hosts; (3) infected male adults do not transmit infections to healthy females; (4) house fly hosts do not become infected; and (5) horizontal transmission occurs when healthy immatures feed on infected larvae in superparasitized hosts (Geden et al., 1995). The intracellular nature of *Nosema* suggested that transmission can occur vertically, either within the egg (cytoplasmic), and/or on the egg surface (per ovum). If per ovum or through ingestion by feeding larvae, parasitoid wasp lines can be cured of the infection by surface sterilization or egg transfer experiments. To establish a parasitoid-*Nosema* model for Nosemosis research, a high-quality reference genome is essential for studying gene expression changes and gene manipulations in *Nosema*. In this study, we sequenced and assembled the *N. muscidifuracis* reference genome in the parasitoid wasp species *M. zaraptor* using PacBio long-read sequencing, developed qPCR assays for accurate quantification of *Nosema* titer, and explored the vertical transmission mechanisms using cytogenetic analyses.

## Results

### *Nosema muscidifuracis* genome assembly and statistics

**Figure 1:**
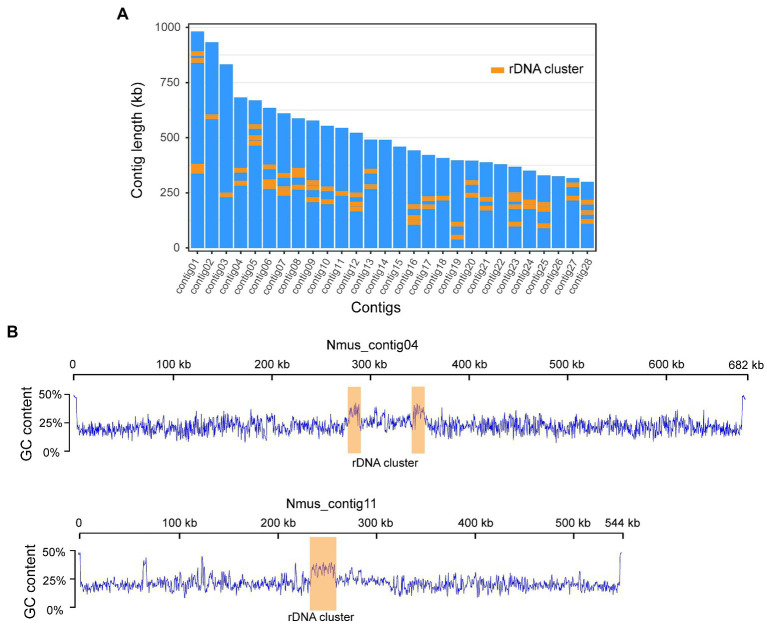
Chromosome level genome assembly of *Nosema Muscidifuracis* showing GC content and rDNA clusters. **(A)** The length of all 28 contigs in *N. Muscidifuracis* genome. A total of 57 rDNA clusters are plotted using orange boxes. **(B)** Plots of GC content along *N. Muscidifuracis* contig04 and contig11 show low average GC-content of the *N. Muscidifuracis* genome and elevated GC content at rDNA clusters.

We sequenced the *M. zaraptor* genome using a combination of PacBio long-read and Illumina 10× Genomics linked-read sequencing technologies in *Nosema*-infected *M. zaraptor* samples (see Materials and methods). A total of 23.7 Gb PacBio Sequel II HiFi reads (61.2-fold coverage of *M. zaraptor* genome), and 63.6 Gb of Illumina linked-reads (54.5-fold coverage) were generated (Supplementary Table S1), resulting in a high-quality assembly of *M. zaraptor* genome. Bioinformatic analyses revealed that symbiotic microbes are among the assembled contigs, including *Nosema muscidifuracis*, a known microsporidian species infecting *M. zaraptor*. *Nosema muscidifuracis* contigs were separated from the host contigs based on a much higher sequencing depth (258.2×; Supplementary Table S2) compared to *M. zaraptor* (61.2×) and a much lower GC content (22.6% compared to 42% in *M. zaraptor*). The drastic differences in sequence coverage and GC content allowed the complete separation of *N. muscidifuracis* contigs from host sequences (Supplementary Figure S1). In addition, *N. muscidifuracis* contigs were aligned to a closely-related, *Nosema*-free *M. raptorellus* genome (Xiong et al., 2021) to confirm the absence of host contaminations. The final assembly of the *N. muscidifuracis* genome contains 14,397,169 bp in 28 contigs, with contig length ranging from 299,473 to 982,164 bp (Figure 1A).

### Assessment of the continuity and completeness of the *Nosema muscidifuracis* genome

The assembled *N. muscidifuracis* contigs had an N50 of 544,348 bp, which is much larger than all published *Nosema* genomes, indicating excellent continuity (Table 1). Nine contigs have regions at termini with elevated GC content (>50%), which are putative telomeric regions, suggesting chromosome-level assembly. To evaluate the completeness of the genome assembly, we aligned 10× Genomics short-read reads using BWA, and 99.42% of the assembled contigs were covered (Supplementary Table S1). The BUSCO completeness score is 97.0%, which is the same as *N. ceranae* assemblies and much higher than other *Nosema* assemblies (Table 1). The assembly statistics indicate that the quality of our assembly is high in both genome continuity and completeness.

**Table 1:** Genome assembly statistics for *Nosema muscidifuracis* and comparison with other microsporidian genomes.

Species (strain)	Accession number	Size (bp)	scaffold/contig N50 (Kbp)	# of scaffolds /contigs	BUSCO complete (fragmented/missing)
*N. muscidifuracis*	This assembly	14,397,169	544.3/544.3	28/28	97.0% (1.2%/1.8%)
*Nosema apis* (BRL01)	GCA_000447185.1	8,569,501	24.3/14.0	554/1,133	75.0% (6.0%/19.0%)
*Nosema ceranae* (BRL)	GCA_004919615.1	8,816,425	177.3/177.3	110/110	97.0% (1.2%/1.8%)
*N. ceranae* (PA08)	GCA_000988165.1	5,690,748	42.6/42.6	536/536	97.0% (1.3%/1.7%)
*N. ceranae* (BRL01)	GCA_000182985.1	7,860,219	2.9/2.9	5,465/5,465	93.9% (3.5%/2.6%)
*Nosema* sp. (YNPr)	NA	3,637,996	12.2/3.8	462/2,272	84.5% (2.7%/12.8%)
*Nosema antheraeae* (YY)	NA	7,100,626	172.2/25.6	202/719	95.3% (1.5%/3.2%)
*Nosema bombycis* (CQ1)	GCA_000383075.1	15,689,776	57.4/6.1	1,607/3,558	83.0% (5.0%/12.0%)
*Nosema granulosis* (Ou3-Ou53)	GCA_015832245.1	8,859,703	12.7/9.4	1,754/2,007	95.9% (1.8%/2.3%)
*Encephalitozoon cuniculi* (GB-M1)	GCA_000091225.2	2,497,519	220.3/218.3	11/12	100.0% (0.0%/0.0%)

### Repeat annotation

In the *N. muscidifuracis* genome, a total of 4,078,013 bp repetitive regions (28.3% of the genome) were identified (Supplementary Table S3). Most repeats belong to the unclassified category (58.8% of total repeats). Among classified repetitive elements, the Gypsy/DIRS1 LTR element is the most abundant, accounting for 37% of all known repeats, and 4.32% of the whole genome. The following classes of repetitive elements account for more than 1% of the *N. muscidifuracis* genome: DNA transposons (3.49%), LINEs (1.68%), and simple repeats (1.71%).

### Noncoding RNA annotation identified 57 rDNA clusters located in the middle of *Nosema muscidifuracis* chromosomes

A total of 327 noncoding RNA (ncRNA) genes were predicted and annotated in *N. muscidifuracis* genome based on the Rfam (Nawrocki et al., 2014) database of RNA families by using the Infernal software package (Nawrocki and Eddy, 2013). The 170 tRNA genes account for 0.09% of the entire genome. We also found seven snRNA associated with U2/U4/U6 small nuclear ribonucleoproteins, and two CD-box, small nucleolar RNA U3, suggesting that the splicing function may be present in *N. muscidifuracis*. The most abundant ncRNA genes in *N. muscidifuracis* are the rDNA genes. We identified 57 complete rDNA clusters encoding 18S/28S ribosomal RNAs (Supplementary Table S4). A hallmark of *N. muscidifuracis* rDNA region is increased GC context (~37%) compared to the genome average (~25%; see Figure 1B). Collectively, the rDNA clustered are more than 206 kbp in length, accounting for 1.4% of the *N. muscidifuracis* genome (Supplementary Table S4).

### Reoccurrence of *Nosema* infection in *Muscidifurax zaraptor* after cured by a combination of heat treatment and Pasteur method

**Figure 2:**
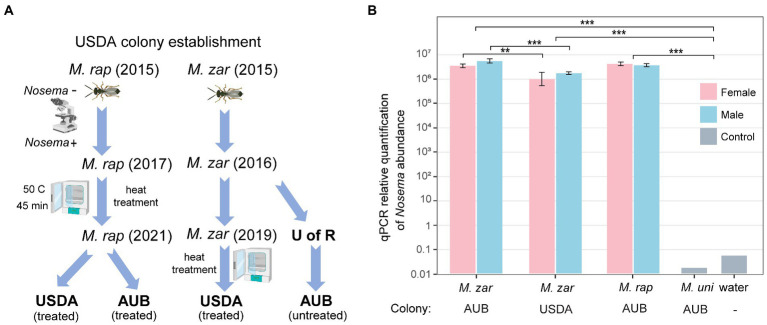
Quantification of the *Nosema* titer in AUB and USDA *Muscidifurax zaraptor* colony. **(A)** The USDA *M. zaraptor* and *M. raptor* colonies were founded in 2015 in Dr. Geden’s laboratory. The USDA *M. raptor* colony was treated using a combination of heat treatment and Pasteur method in 2017, resulting in a *Nosema*-free colony confirmed by microscopic examination. The AUB *M. zaraptor* was derived from a colony from the University of Rochester (U of R), which was never treated. The USDA *M. zaraptor* colony was cured for *Nosema* infection using a heat treatment approach in 2019 (Geden et al., 2019). **(B)** Results of qPCR quantifications of *Nosema muscidifuracis* in *M. zaraptor* and *M. raptor*. Female samples were plotted using pink bars and male samples were plotted in blue. Negative control (water) and *Nosema*-free wasp *M. uniraptor* were included as controls. Statistical significance was assessed by *t*-test (*, *p* < 0.05; **, *p* < 0.01; ***, *p* < 0.001).

The genome assembly used *M. zaraptor* in the AUB colony, which has never been treated for *Nosema* (Figure 2A). In 2019, treatment to eliminate *Nosema* was performed at the USDA colony, using a heat incubation approach (Boohene et al., 2003). The *M. raptor* USDA colony was established in 2015 from *Nosema*-free founders based on microscopic examination (Zchori-Fein et al., 1992). The recurrence of *Nosema* infection was cured in 2017 using an extensive treatment procedure described in Materials and methods (summarized in Figure 2A). To determine whether the treatment has effectively eliminated or reduced the *Nosema* infection, we designed PCR primer sets (Supplementary Table S5) to target the 18S rDNA genes with sequence information in our genome assembly. The *Nosema* titers in the AUB (Ct values 10.2~11.7) and USDA (Ct value 12.1~13.8) *M. zaraptor* samples were extremely high compared to the control uninfected *Muscidifurax uniraptor* samples (Ct value >38; *p* < 0.001, *t*-test), indicating heavy infection in both colonies (Figure 2B). The relative abundance of *Nosema* in AUB samples is significantly higher than the USDA colony for both females (2.8-fold; *p* < 0.01, *t*-test) and males (3.3-fold; *p* < 0.001, *t*-test), suggesting a significant reduction of the *Nosema* load in treated wasps (Figure 2B). The results indicated that heat treatment reduced the *Nosema* titer in a short time but failed to eradicate the *Nosema*, and the infection came back and reached high titer rapidly. For the *M. raptor* samples, the relative abundance of *Nosema* (Ct values 10.8~11.7) was also high compared to the *Nosema*-free *M. uniraptor* samples (*p* < 0.001, *t*-test), which is comparable to AUB *M. zaraptor* samples (Figure 2B). It is clear the *Nosema* has rebounded in the heat-treated line, either due to incomplete elimination or cross-infection from infected wasps.

### Comparative genomic analysis of *Nosema muscidifuracis* with *Nosema ceranae* and an outgroup microsporidian *Encephalitozoon cuniculi*

**Figure 3:**
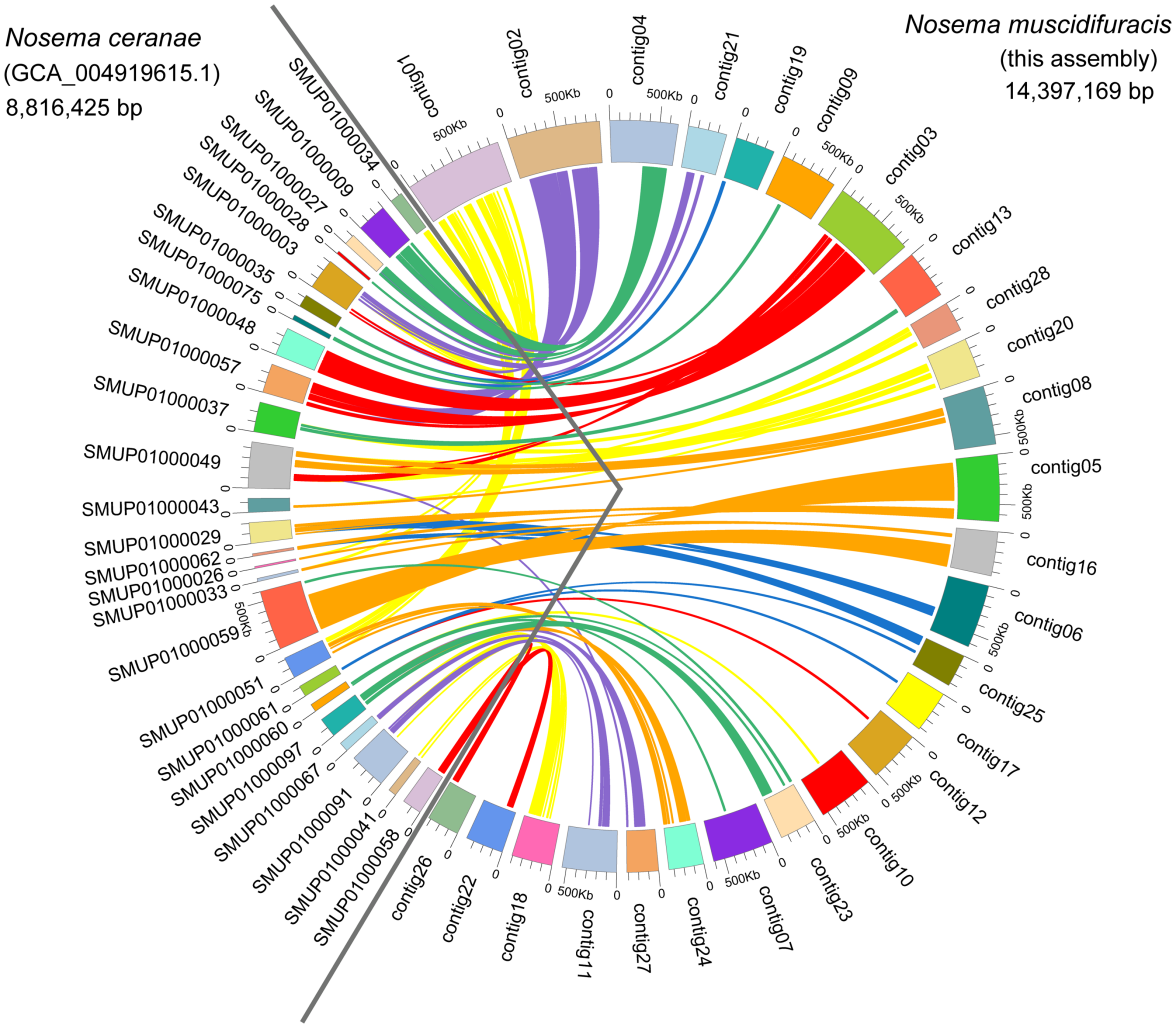
Genome comparisons between *Nosema muscidifuracis* and *Nosema ceranae*. A total of 26 contigs of *N. muscidifuracis* (93.4% of assembly in this research) show a one-to-one relationship with 25 scaffolds in the *N. ceranae* genome (54.1% of assembly GCA_004919615). The scaffolds on the left of the circle represent *N. ceranae* scaffolds, and the contigs on the right represent *N. muscidifuracis* contigs.

We identified 2,782 protein-coding gene models using Fungal Genome Annotation Pipeline (FunGAP; Min et al., 2017), with RNA-seq read support (Supplementary Table S6). The number of protein-coding genes we annotated for *N. muscidifuracis* is close to that of *N. ceranae* (*N* = 2,905; Cornman et al., 2009) and *N. apis* (*N* = 2,764). Comparative genomic analysis revealed that 26/28 *N. muscidifuracis* contigs have syntenic regions in the *N. ceranae* genome based on protein-coding genes (Figure 3). There is a moderate level of conservation in gene order, with many genome rearrangement events (Figure 3). When an outgroup microsporidian species was compared, all 11 chromosomes in *E. cuniculi* (Katinka et al., 2001) have syntenic regions in *N. muscidifuracis*, which mapped to 24 *N. muscidifuracis* contigs (Supplementary Figure S2).

### Genome annotation reveals extensive gene duplication events in *Nosema muscidifuracis*

**Figure 4:**
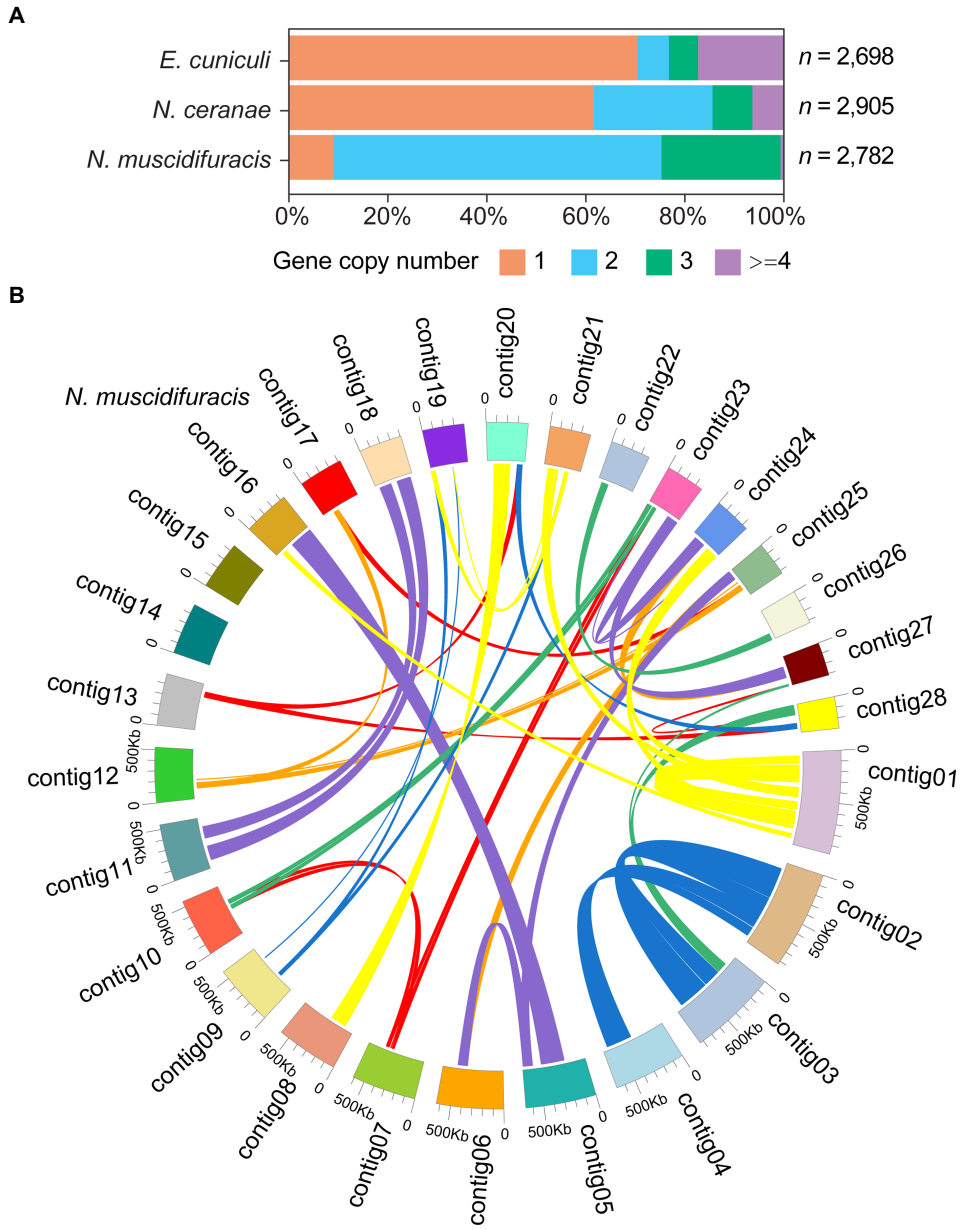
Histograms of gene copy number in *Nosema muscidifuracis*, *Nosema ceranae,* and *Encephalitozoon cuniculi* genomes and self-circos plot of 28 contigs in *N. muscidifuracis*. **(A)** Proportion of annotated genes (*x*-axis) with different gene copy number in the genomes of *N. muscidifuracis, N. ceranae,* and *E. cuniculi* (*y*-axis). **(B)** The self-circos of *N. muscidifuracis* genome shows the linked relationship of 28 contigs.

In the *N. muscidifuracis* genome, the majority of genes were duplicated with two or more copies (*N* = 2,526 genes in 1,147 paralogous groups). In sharp contrast, only a small number of genes (*N* = 256) are single-copy (Figure 4A). This is not the case in *N. ceranae* or *E. cuniculi* genomes (Figure 4A). The pattern is very complex (Figure 4B), indicating extensive duplications and rearrangements within the genome. For *N. muscidifuracis* with two copies, we aligned all 919 pairs and found that 859 of them are the same length, and only 60 pairs have different gene sizes. The average pairwise identity for the 859 gene pairs with the same length is 99.7%, and that of the remaining 60 gene pairs is 94.1%. This indicates relative recent duplications in *N. muscidifuracis*. We computed the number of substitutions for the 859 gene pairs with the same size (average gene length = 1,027 bp), and on average, 1.66 synonymous and 1.09 nonsynonymous substitutions were observed (2.75 total). We next examined the syntenic pattern between homologous genes within *N. muscidifuracis*. A self-circos plot (syntenic relationship among contigs determined by homolog of gene models) did not reveal whole contig duplications, but rather extensive duplications and rearrangements across the genome (Figure 4B). The majority of the duplicated gene pairs are on different contigs, except for 66 gene pairs on contig01 (Figure 4B). Contig01 is the largest contig/chromosome (982 kb in length), with duplicated regions on contig16, contig21, and contig24, with short to moderate stretches of synteny (Figure 4B). To check whether the duplication events have an impact on the genome size, we estimated the genome size of *N. muscidifuracis* using a k-mer approach (see Materials and methods), and the estimated genome size is approximately half of the assembled size (7,040,619 bp) with an estimated heterozygosity of 0.9%, which is consistent with the extensive duplication events we observed.

**Figure 5:**
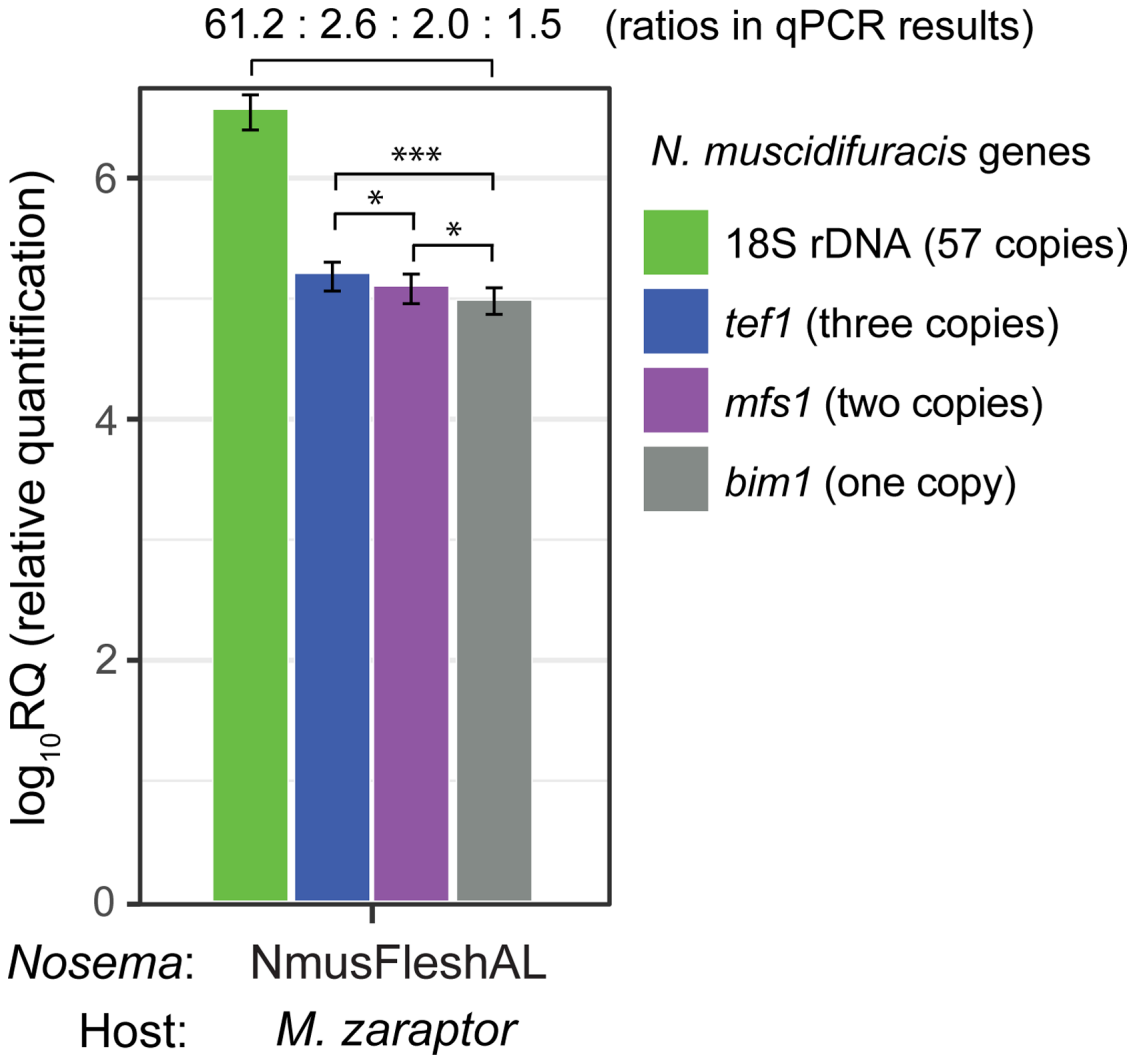
Quantification and confirmation of gene copy number in *Nosema muscidifuracis* using quantitative PCR approach. Results of qPCR analysis of 18S rDNA gene (57 copies in the genome, in green), protein-coding genes *tef1* (three copies, in blue), *mfs1* (two copies, in purple), and *bim1* (single-copy, in gray). NmusFleshAL: *Nosema*-infected *Muscidifurax zaraptor* reared using flesh fly pupae at Auburn, Alabama. Statistical significance was assessed by *t-test* (*p < 0.05; ***p < 0.001).

We examined the read depth in single-copy gene regions and duplicated gene regions. There are significant differences in depth between single-copy genes and genes with two copies. For single-copy genes, the average coverage depth is 44.7 with a standard error of 1.6. In duplicated gene regions, the average coverage total depth is 72.0 with a standard error of 0.7, which is significantly higher than single-copy genes (*p* < 2.2 × 10^−16^, Mann–Whitney U test). To further confirm the gene duplication events, we quantified the relative abundance of a single-copy gene *bim1* (microtubule binding protein BIM1), a two-copy genes *mfs1* (major facilitator superfamily 1 nucleoside transporter), and a three-copy gene *tef1* (translation elongation factor 1 alpha), and compared them with the 18S rDNA gene (Figure 5). The *tef1* relative abundance is significantly higher than *mfs1* (*p* < 0.05, *t*-test), and *mfs1* has significantly higher abundance that the one-copy *bim1* gene (*p* < 0.05, *t*-test; Figure 5). The read depth and qPCR results confirmed the copy number differences of the *N. muscidifuracis* genes.

### Cytogenetics of reproductive tissues

**Figure 6:**
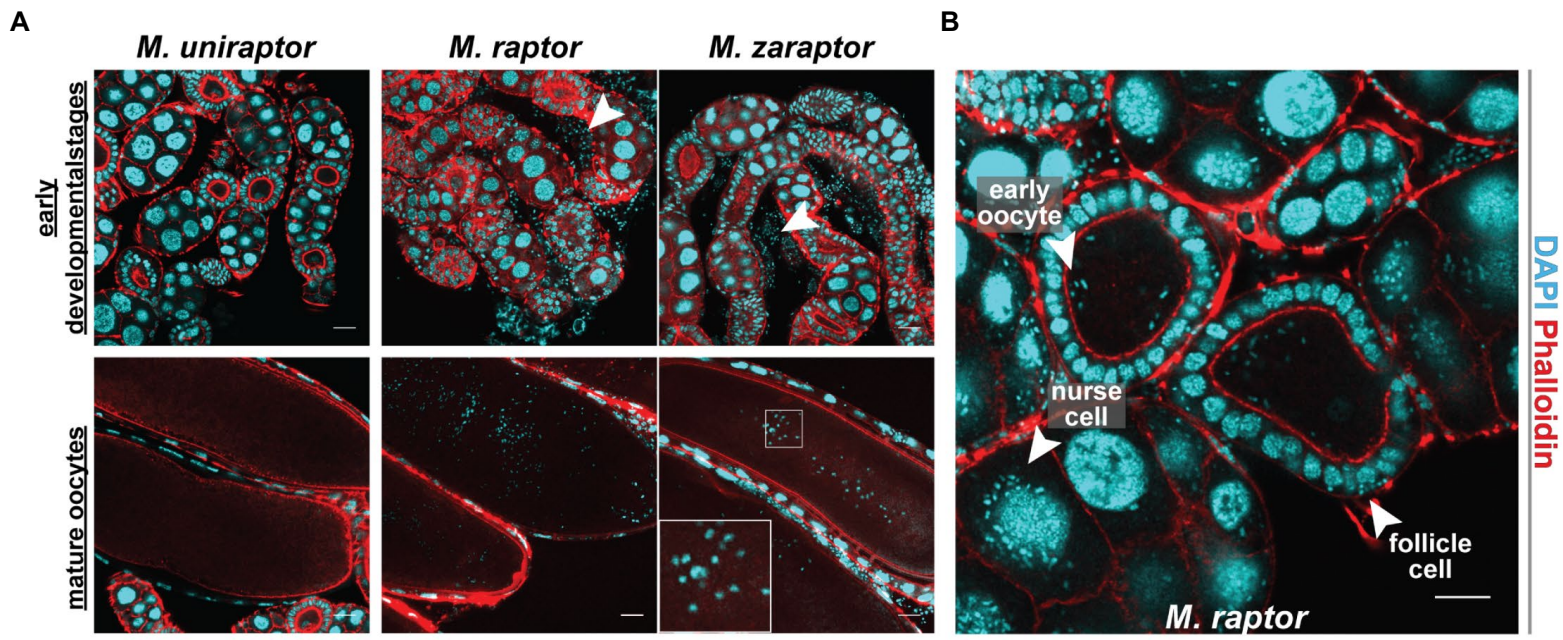
Cytogenetic analysis of *Nosema muscidifuracis* distribution in developing and mature oocytes of three *Muscidifurax* species. **(A)** Staining of ovaries from adult female *Muscidifurax uniraptor*, *Muscidifurax raptor*, and *Muscidifurax zaraptor* (from *left* to *right*) showing early developmental stages (*top*) and mature ovaries (*bottom*). *Red*, actin staining using Rhodamine Phalloidin. *Blue*, DNA staining using DAPI. The length of the scale bar is 20 μm. **(B)** Staining showing the distribution of *N. muscidifuracis* in *M. raptor* early oocyte, follicle cells, and nurse cells (white arrows). The length of the scale bar is 20 μm.

*Nosema muscidifuracis* shows evidence of vertical transmission. We therefore investigated the ovaries of three species, *Muscidifurax zaraptor* and *M. raptor*, which are infected with *Nosema*, and the closely-related species *M. uniraptor*, which is *Nosema*-free despite being reared in the same research environment. Staining with the DNA intercalating agent DAPI revealed puncta of ~2 μM in diameter, much smaller than nurse cell or follicle cell nuclei, in ovarioles from *M. zaraptor* and *M. raptor* but not *M. uniraptor*. These puncta, which are consistent in appearance with previous observations of *N. muscidifuracis* (Pei et al., 2021), are associated with developing egg chambers at all stages and can be readily distinguished inside late-stage oocytes (Figure 6). Infected ovarioles also demonstrate signs of infection in other cell types, including nurse cells and follicle epithelial cells (Figure 6). This confirms heavy infections of microsporidia in the ovaries, and likely supports cytoplasmic transmission of the microbe through eggs.

## Discussion

### A high-quality genome assembly of *Nosema muscidifuracis*

*Nosema* (Microsporidia: Nosematidae) is one of the most widespread unicellular parasites belonging to the microsporidia group. Previously, microsporidia were classified as protozoan, but recently they were recognized as a group of fungi. As a genus in microsporidia, *Nosema* infect insects and other arthropods using a specialized organelle known as the polar filament, which is coiled inside its spores. Compared to other microsporidians, *Nosema* has a much lower GC content, which makes it very difficult to assemble its genome at high levels of completeness and continuity. In this study, we sequenced and annotated the genome of *Nosema muscidifuracis*, which is the *Nosema* species infecting the parasitoid wasp species *Muscidifurax zaraptor* and *M. raptor*. This is the first genome assembly of *N. muscidifuracis*, with the highest completeness (97.0% BUSCO score) and continuity (contig N50 = 544 kb) among all available *Nosema* genomes (Table 1). The high quality and well annotated genome of *N. muscidifuracis* is crucial for the comparative genomic analysis and evolutionary study in *Nosema*, and will facilitate future genome manipulation in this fungal pathogen to control Nosemosis.

### Variation in genome size among *Nosema* species

Microsporidia usually have a severely reduced genome, such as a 2.5 Mb genome in *E. cuniculi* (Katinka et al., 2001; Peyretaillade et al., 2009). In *Nosema*, the smallest reported genome is *Nosema* sp. YNPr (3.6 Mb), which infects the cabbage butterfly *Pieris rapae* (Xu et al., 2016). However, the BUSCO completeness score was 84.5%, raising the question of whether sizeable numbers of genes were missed in that assembly (Table 1). The smaller genome may be partly due to incomplete genome assembly, or loss of some conserved genes in microsporidia. Four *Nosema* species have a reported genome size of 7~9 Mb, including 8.8 Mb in *N. ceranae* BRL (Huang et al., 2021), 8.6 Mb in *N. apis* (Chen et al., 2013), 7.8 Mb in *N. antheraeae* infecting the Chinese tussar moth *Antheraea pernyi* (Li et al., 2017), and 8.6 Mb *N. granulosis* infecting the Amphipod *Gammarus duebeni* (Cormier et al., 2021). The 15.7 Mb *N. bombycis* (Pan et al., 2013) and 14.4 Mb *N. muscidifuracis* (this research) assemblies are a bit less than doubled genome size of other *Nosema* species (Table 1), indicating changes in the genome size in *Nosema*. A larger genome harbors more genes for adapting to different conditions and host environments, but this increase in capacity could also be achieved by polyploidy, which is quite common in intracellular parasites (Nakabachi and Moran, 2022). The evolution of genome size in *Nosema* and other microsporidia warrants further study. Furthermore, little is known about the sexual cycle of *Nosema*, and how this may relate to life cycle changes in ploidy levels.

### Extensive gene duplication events in *Nosema muscidifuracis*

We observed a unique feature in the *N. muscidifuracis* genome, which is the majority of protein-coding genes have two copies (66.2% of genes) or multiple copies (8.1%). One possible reason for this observation is polyploidy, which is a phenomenon that organisms possess more than two complete sets of chromosomes in individual cells (Van de Peer et al., 2017). However, the duplication of genomic regions in *N. muscidifuracis* is not consistent with complete chromosome duplication (Figure 4). Based on our results, the most plausible scenario would be extensive gene duplications affecting multiple regions in the genome. In fungi, alternating haploid and diploid phases are present in many species (Valero et al., 1992), and it is not clear whether such a process exists in *Nosema*. Polyploidy has been suggested in natural *N. ceranae* population (4 N or more), based on the distribution of genetic variation (Pelin et al., 2015). A recent study of multiple fungal genomes identified that most zoosporic fungi, including microsporidia, are diplontic (diploid-dominant) based on SNP density (Amses et al., 2022). The density of pairwise substitutions in *N. muscidifuracis* duplicated genes is 2.68 × 10^−3^ in this study, which would be classified as diplontic according to the criteria for diploid genomes (mean = 2.04 × 10^−3^) in Amses et al. (2022). However, not all *N. muscidifuracis* genes are duplicated (only 66%), and our long-read assembled results do not support complete chromosome duplication. It is likely to be a complex history of gene duplication and rearrangement in *N. muscidifuracis* evolution, which expanded its genome size and resulted in the gene copy number profile we observed in this study.

### Transmission of *Nosema* in parasitoid wasps

Transovarial transmission of unicellular parasites has been observed and reported in microsporidia (Becnel, 1994; Terry et al., 1997; Dunn et al., 2001; Pei et al., 2021). In this study, we established vertical transmission of *N. muscidifuracis* in the parasitoid wasps *Muscidifurax zaraptor* and *M. raptor* through straining experiments in the ovaries of infected females. However, whether this is the exclusive or primary mode of transmission remains to be determined. In addition, maternal transmission by injection of microsporidia during stinging of the host is also possible, which could also result in transfer between lineages when two females parasitized the same host. Additional studies are needed to characterize the transmission mechanism(s), which will be relevant to attempts to maintain *Nosema*-free colonies of these biological control agents.

### Toward an ultimate cure for Nosemosis

Pest flies can cause a huge economic loss on livestock operations. The impact of stable flies on feeder cattle dropped feed efficiency by 10% to 15%, and gains were reduced by 0.2 to 0.5 pounds per day (Taylor et al., 2012). When dairy cattle spend their energy getting the flies off their legs, bunching in a corner, or not eating, milk production can be reduced by almost 2.2 pounds per day. It is estimated that stable flies can cost the livestock industry $2.2 billion per year. Parasitoid wasp species in the genus of *Muscidifurax* are excellent biological control agents for dipteran filth flies, including house fly, stable fly, horn fly, black dump fly, and flesh fly (Petersen and Currey, 1996; Geden and Hogsette, 2006; Geden and Moon, 2009; Machtinger and Geden, 2018). These parasitoids are more environment-friendly and sustainable, and host flies are not known to develop resistance to them, as they do with chemical insecticides (Oyarzun et al., 2008; Scott et al., 2013). The production of parasitoids is affected by the *Nosema* disease caused by persistent infection of *N. muscidifuracis*, which significantly reduces fitness and fecundity (Geden et al., 1995). A heat shock treatment approach is effective in controlling *Nosema* disease, resulting in a 100% cure rate and significantly improved fitness (Boohene et al., 2003). In 2019, the USDA *M. zaraptor* colony was cured by heat treatment. However, the *Nosema* bounced back to a high level within a short period of time, based on microscopic inspection, and qPCR quantification. The *Nosema* titer in the USDA *M. zaraptor* is about a third of the untreated lines (AUB), although the titer is high in both colonies. The USDA *M. raptor* colony was started from *Nosema*-free founders based on microscopic examinations. However, infection was observed after 1 year, and the *M. raptor* colony was treated using a series of heat treatment procedures in 2017. Parasitoids from the colony appeared to still be uninfected after 3 months, but infection reappeared after several years of rearing without regular inspection or further treatment for infection. These results demonstrate that visual inspection for patent infections is an insufficiently sensitive metric for eliminating *Nosema* disease from colonies of *M. raptor*. In the qPCR results, the average Ct value was 11.23, which is orders of magnitude higher than uninfected controls (Ct value = 38; Figure 2B). Therefore, the carefully designed extensive heat treatment approach failed to eradicate *Nosema*, and the infection came back and reached high titer. The vertical transmission we showed may play a role in the rapid reoccurrence of *Nosema* because some spores could escape the treatment and be transmitted in the eggs. To combat this highly infectious parasite, molecular approaches, such as RNA interference or *Nosema* genome manipulation, need to be considered. Our high-quality genome assembly and annotation serve as a first step to providing the necessary genome toolkit for these approaches, and for a better understanding mechanisms and etiology of Nosemosis.

## Materials and methods

### Sample source and insect rearing

Three *Muscidifurax* species, *M. zaraptor*, *M. raptor*, and *M. uniraptor*, were used in this study. The source of *M. zaraptor* was from two independent colonies, and both of them were derived from the same USDA colony maintained by Dr. Chris Geden’s laboratory, which was originally collected in 2015 from dairy farms in Minnesota, Nebraska, and California. The USDA *M. zaraptor* colony is maintained in the Geden laboratory at the Center for Medical, Agricultural and Veterinary Entomology, USDA Agricultural Research Service (USDA-ARS, Gainesville, FL, United States). An attempt was made to cure the colony for *Nosema* infection in 2019 using a heat shock treatment approach (Geden et al., 2019). The *M. zaraptor* colony maintained in the Wang laboratory (AUB colony) at Auburn University College of Veterinary Medicine (Auburn, AL, United States) was obtained from the University of Rochester in 2019, which was derived from the USDA colony infected with *N. muscidifuracis*. The *M. uniraptor* colony at AUB was also obtained from the University of Rochester in 2019, and it is *Nosema*-free. The *M. raptor* colony maintained in the Wang laboratory at AUB was derived from a *Nosema*-cured colony treated in 2017 in the Geden laboratory (see Materials and methods). All AUB colonies were maintained on commercial flesh fly (*Sarcophaga bullata*) pupae (Ward’s Science, Rochester, NY, United States) at a constant temperature of 25°C and 24 h constant light in the Wang laboratory. The USDA colonies were maintained on housefly (*Musca domestica*) pupae in the Geden laboratory (Gainesville, FL).

### High molecular weight DNA extraction, PacBio CCS library preparation and sequencing

High molecular weight (HMW) genomic DNA (gDNA) was extracted from *M. zaraptor* whole-body samples collected 24 h after eclosion from the AUB colony using Genomic-tip 20/G kit (Qiagen, MD, United States). The DNA concentration was measured on a Qubit 3.0 Fluorometer instrument (Thermo Fisher Scientific, MD, United States). The gDNA quality and the size distribution were assessed on an Agilent TapeStation 4200 machine (Agilent Technologies, CA, USA) with the genomics screentapes. A total of 10 μg high-quality *M. zaraptor* HMW gDNA was sheared into 20 Kb fragments. After end-repair and ligating the specific adapter oligos, the DNA fragments were annealed with sequencing primer v2 and Sequel II DNA Polymerase, bound to the SMRTbell templates, and the library was prepared using the SMRTbell Template Prep kit v2 with the CCS HiFi Library construction protocol (Pacific Biosciences, CA, United States) at the HudsonAlpha Genome Sequencing Center (HGSC, Huntsville, AL, United States). The concentration and the size distribution for the prepared library were determined on LabChip GX Touch HT (PerkinElmer, MA, United States), and sequenced on a PacBio Sequel II System at HGSC (Supplementary Table S1).

### 10× genomics linked-read library construction and Illumina sequencing

HMW gDNA from AUB *M. zaraptor* was diluted to ~0.8 ng/uL with EB buffer through a series of dilutions, with concentrations determined by Qubit 3.0 Fluorometer (Thermo Fisher Scientific, United States). The Chromium Genome Reagent Kit v2 (10× Genomics Inc., CA, United States) was used for linked-read library preparation, according to the manufacturer’s instructions. The genome chip was loaded with diluted denatured gDNA, sample master mix, and gel beads following the protocol. Gel Bead-In-EMulsions (GEMs) were generated using a 10× Chromium Controller. After the incubation and cleanup of the obtained GEMs, Chromium i7 Sample Index was ligated and served as the library barcode to provide linked information. The size distribution of the prepared library was assessed using Agilent TapeStation 4200 (Agilent Technologies, CA, United States), final library quantity was checked with Qubit 3.0 Fluorometer (Thermo Fisher Scientific, United States). After quality control, the 10× Genomic library was sequenced on an Illumina NovaSeq 6000 machine.

### Assembly of the *Nosema muscidifuracis* genome

*De novo* genome assembly for the *M. zaraptor* genome was performed with 23.7 Gb PacBio HiFi reads (377.2 Gb raw reads) using dedicated long-read assemblers hifiasm v0.13 (Cheng et al., 2021). The 10× Genomics reads were aligned to the assembled contigs using the LongRanger pipeline v2.1.6 (McKenna et al., 2010). The identity of *M. zaraptor* contigs was determined by coverage depth (54.5X Illumina read depth) and homology to closely-related *M. raptorellus* with a high-quality reference genome available (Xiong et al., 2021). Contigs from six microbial species were also detected in the hifiasm assembly, including five bacterial species and *N. muscidifuracis*. A total of 30 *N. muscidifuracis* PacBio HiFi contigs were identified based on coverage depth, GC content, and CpG percentages (Wang et al., 2019). *De novo* assembly of 10× Genomics data was performed using Supernova v2.1.1 with default parameters (Weisenfeld et al., 2017). Overlap of HiFi contigs and 10× scaffolds were detected by quickmerge v0.3.0 (Chakraborty et al., 2016). The 30 *N. muscidifuracis* contigs were merged into 28 contigs based on manual inspection of the contig overlap.

### Assessment of *Nosema* genomes

The final genome completeness of *N. muscidifuracis* was assessed by BUSCO (Benchmarking Universal Single-Copy Orthologs) v5.3.2 (Seppey et al., 2019). The BUSCO scores were computed using microsporidia_odb10 with a total of 600 orthologs. The BUSCO scores were also computed for an outgroup microsporidian species, *Encephalitozoon cuniculi* (Katinka et al., 2001; Peyretaillade et al., 2009), as well as eight other *Nosema* species/strains, including *N. ceranae* BRL 01 (Cornman et al., 2009), *N. ceranae* PA08 (Pelin et al., 2015), *N. ceranae* BRL (Huang et al., 2021), *N. apis* BRL 01 (Chen et al., 2013), *N. bombycis* CQ1 (Pan et al., 2013), *N. granulosis* Ou3-Ou53 (Cormier et al., 2021), *N. antheraeae* YY (Li et al., 2017), and *Nosema* sp. YNPr (Xu et al., 2016; Table 1).

### Genome size estimation

Illumina short-reads aligned to *N. muscidifuracis* assembly were utilized to estimate the genome size. Low-quality bases and adapter sequences were trimmed using Trimmomatic version 0.39 (Bolger et al., 2014), with the parameters “ILLUMINACLIP:adapter:2:30:10 LEADING:3 TRAILING:3 SLIDINGWINDOW:4:15 MINLEN:60.” High-quality trimmed reads were used for multiple k-mer counting using Jellyfish version 2.3.0 (Amoroso, 1968) with parameters count “-m 25 -s 20G -t 48.” The genome size and heterozygosity were estimated using GenomeScope (Vurture et al., 2017).

### Repeat annotation

Before gene prediction, repeat annotation was performed to identify the repetitive elements in *N. muscidifuracis* genome. We first constructed a *de novo N. muscidifuracis* repeat database using RepeatModeler v2.0.1 (Flynn et al., 2020), which provided a list of repeat family sequences. The repeat-identifying was implemented by three complementary computational programs, RECON v1.0.8 (Bao and Eddy, 2002), RepeatScout v1.0.5 (Price et al., 2005), and Tandem Repeats Finder (TRF; Benson, 1999). Based on the transposon element library, the homologous repeats and low-complexity DNA sequences were masked using RepeatMasker v4.1.1 (Tarailo-Graovac and Chen, 2009) with RMBlast v2.10.0 sequence search engine (Supplementary Table S3).^1^

### Noncoding RNA annotation

Noncoding RNAs (ncRNAs) were predicted using the Infernal software version 1.1.2 (Nawrocki and Eddy, 2013) based on the multiple sequence alignments using the covariance models in the Rfam database (Nawrocki et al., 2014). Before prediction, the esl-seqstat program was used to determine the total database size for the *N. muscidifuracis* genome. The ncRNAs in the *N. muscidifuracis* genome were predicted and annotated using the cmscan program in Infernal software (Nawrocki and Eddy, 2013) with RNA families in the Rfam (Nawrocki et al., 2014) database (Supplementary Table S4).

### *Nosema* inspection and treatment procedures

Colonies of *M. raptor* were established in 2015 from parasitoids collected from dairy farms in Florida, Minnesota, Nebraska, and California, and samples of parasitoids were examined visually for *Nosema* infection by crushing wasps in a drop of sterile water on a microscope slide and examining at 400X for the presence of spores (Zchori-Fein et al., 1992). None of the specimens examined at the time showed patent infections. After 1 year in colony, 100% of examined parasitoids had patent infections, indicating that low-level infections had escaped detection at the time of colony founding. In 2017, colonies were heat-treated by exposing multiple cohorts of newly parasitized house fly pupae to 50°C for 45 min and holding them for adult emergence (Boohene et al., 2003). Emerged adults were examined visually for infection as before, and progeny from cohorts that were *Nosema*-free based on visual inspections were used to start new colonies. Examination of these colonies after three additional generations indicated a resurgence of infection in three out the four strains. A final attempt was made to eliminate *Nosema* disease from the colonies by first subjecting three successive generations of parasitoids from the four strains to heat treatment as before and holding them for adult emergence. This procedure was conducted with three separate lineages of each of the four strains. Parasitized pupae were then held in individual gelatin capsules for emergence. Pairs of parasitoids (*n* = 100 pairs for each strain) were then held with 70 house fly pupae/pair for 3 days, then given another set of 70 for three more days. Each female was examined individually for the presence of spores; it was expected that 6 days would be sufficient time for infections to be evident by visual inspection. Progeny of any females with patent infections were discarded, and the parasitized pupae from females without patent infections were pooled to start a new colony.

### Genomic DNA extraction and *Nosema* titer determination using quantitative PCR

To determine the levels of *Nosema* infection in the *Nosema*-cured *M. raptor* AUB colony, *Nosema*-infected *M. zaraptor* AUB colony and *Nosema*-cured *M. zaraptor* USDA colony, we extracted genomic DNA from 24-h adult male and female samples with three replicates per sex using AllPrep DNA/RNA Mini Kit (Qiagen, MD). Three primer sets were designed to target the different regions of the 18S rDNA gene in *N. Muscidifuracis* using Oligo 7 primer analysis software (Molecular Biology Insights Inc., Cascade, CO, United States; Supplementary Table S5). Primers were synthesized at Eurofins Genomics LLC (Louisville, KY, United States). All primer sets were evaluated by PCR using the *M. zaraptor* DNA template, with *Nosema*-free DNA as a negative control, followed by electrophoresis on 2% agarose gel (Supplementary Figure S3). The PCR experiments were performed with Q5 High-Fidelity 2x Master Mix on an Eppendorf Mastercycler Pro PCR machine (Eppendorf North America, Enfield, CT, United States). The thermal cycling protocol for the primers was as follows: initial denaturation step at 98°C for 30 s, followed by 30 cycles of initial denaturation at 98°C for 10 s, annealing at 55°C for 30 s, and extension at 72°C for 15 s. The qPCR reaction was conducted in a 20 μL system using Luna® Universal qPCR Master Mix (New England BioLabs, Ipswich, MA, United States). Each reaction contained 10 μL of Luna Universal qPCR Master Mix, 8 μL of nuclease-free water, 0.5 μL of forward primer and 0.5 μL reverse primer (10 μmol/L), and 1 μL of DNA template. The Bio-Rad C1000 Touch Thermal Cycler with CFX96 Real-Time PCR Detection Systems (Bio-Rad Laboratories, Hercules, CA, United States) was used to conduct qPCR experiments with the SYBR scan mode using the *Nosema* 18S NP2 primer set (Supplementary Table S5). The relative quantification method was applied to determine the *Nosema* abundance in *M. raptor* AUB colony and *M. zaraptor* from both AUB and USDA colonies. The thermocycling conditions for the qPCR assays were 95°C for 60 s, followed by 40 cycles at 95°C for 15 s and 50°C for 30 s.

### RNA sample quality control, RNA-seq library preparation and sequencing

Adult male and female *M. zaraptor* were collected 24 h after eclosion from both the USDA colony and AUB colony in the Wang laboratory. Total RNA was extracted from the adult whole-body samples in three biological replicates for each sex. RNA extractions were performed with AllPrep DNA/RNA Mini Kit (Qiagen, MD, United States) following the manufacturer’s protocol. The RNA yield was quantified using a Qubit 3.0 Fluorometer instrument (Thermo Fisher Scientific, MD, United States), followed by a quality check on an Agilent TapeStation 4,200 Bioanalyzer (Agilent Technologies, CA, United States). For RNA-seq library preparation, 1 μg of total RNA was used as input for all samples. To remove the abundant rRNA (ribosomal RNA) in the sample, an rRNA removal protocol was performed using the NEBNext rRNA Depletion Kit (New England Biolabs, MA, United States). The remaining mRNA was used for RNA-seq library construction using NEBNext Ultra II Directional RNA Library Prep Kit (New England Biolabs, MA, United States) with the manufacturer-provided protocol. After quality control, the RNA-seq library was sequenced on an Illumina NovaSeq6000 platform (Supplementary Table S6).

### RNA-seq data processing and gene annotation

On average, 128 million 150-bp reads were generated for each of the 12 *M. zaraptor* RNA-seq libraries. The raw RNA-seq data was checked for sequencing quality using FastQC (Andrews et al., 2010). Adapter sequences and low-quality bases in the paired-end RNA-seq reads were trimmed with Trimmomatic v0.39 (Bolger et al., 2014). A total of 6.2 million non-rDNA reads mapped uniquely to the *N. muscidifuracis* were used for *de novo* transcriptome assembly by Trinity v2.4.0 (Haas et al., 2013). Protein-coding genes were predicted and annotated in the *N. muscidifuracis* genome assembly using Fungal Genome Annotation Pipeline (FunGAP; Min et al., 2017), and filtered RNA-seq reads as input. FunGAP masked the repeats in the genome and assembled the RNA-seq reads. Augustus (Stanke et al., 2006) was used for gene model prediction with species parameter “--augustus_species encephalitozoon_cuniculi_GB,” which is the closest to *Nosema*. The microsporidian protein database used by FunGAP was downloaded by “download_sister_orgs.py” script in FunGAP.

### Comparative genome analysis

To compare the genomes of *N. muscidifuracis* and its closely-related species *N. ceranae,* MCscanX (Wang et al., 2012) was used to perform synteny analysis and identify syntenic blocks between these genomes based on core orthologous gene sets identified using BlastP with default settings (E-value ≤ 1e−5; minimum number of genes in a syntenic block ≥ 5). The genome and annotation files of *N. ceranae* (BRL strain) were downloaded at NCBI Assembly with the accession number GCA_004919615.1. The genomic circle of collinearity was visualized in Circos (Krzywinski et al., 2009). To check the location of duplicated genes in *N. muscidifuracis*, we detected and plotted the collinear blocks within the *N. muscidifuracis* genome using MCscanX (Wang et al., 2012). The duplicated genes were aligned using mafft software (version 7.475; Katoh and Standley, 2013). The identity of the two sequences for each gene pair was computed by the Needle program with the default parameters (Gap penalty = 10, Extend penalty = 0.5) using the Needleman-Wunsch algorithm (Needleman and Wunsch, 1970). The numbers of synonymous (dS) and non-synonymous (dN) substitutions between two sequences were calculated using the alignments by KaKs_Calculator software (version 2.0) with γ-YN method (Yang and Nielsen, 2000; Wang D. et al., 2009; Wang D.-P. et al., 2009; Wang et al., 2010).

### Confirmation of gene copy number differences using qPCR

We selected one single-copy gene *bim1*, *mfs1* with two homologous, and *tef1* with three homologous copies in *N. muscidifuracis* genome for qPCR validation. The Oligo 7 primer analysis software (Molecular Biology Insights Inc., Cascade, CO, USA) was used to design the primer sets targeting the three selected protein-coding genes. The primers used for *bim1* are 5′-GTAGAAGAGAATTGCTTGAATG-3′ and 5′-ACTCATACTCTGATGAAGGATTT-3′, the primers used for *mfs1* are 5′-TTTAGCCACAAAATTATGTCC-3′ and 5′-ATGTTAAATACTTGTGCTCT-3′, and the primers used for *tef1* are 5′-GCTGCTGAAAATAACAAGTCT-3′ and 5′-GCTGGTACAATAACTACACCT-3′. All primers were synthesized by Eurofins Genomics LLC (Louisville, KY, United States). Before the qPCR experiments, we checked the size of PCR products and the amplification efficiency using 2% agarose gel electrophoresis. The qPCR experiments were performed using Luna® Universal qPCR Master Mix (New England BioLabs, Ipswich, MA, United States) on a Bio-Rad C1000 Touch Thermal Cycler with CFX96 Real-Time PCR Detection Systems (Bio-Rad Laboratories, Hercules, CA, United States). The 20 μL reaction system consisted of 10 μL of Luna Universal qPCR Master Mix, 0.5 μL of forward primer (10 μmol/L), 0.5 μL of reverse primer (10 μmol/L), 8 μL of nuclease-free water, and 1 μL DNA template (*M. zaraptor* DNA samples extracted from AUB colony). The qPCR reaction conditions were 95°C for 60 s, followed by 40 cycles at 95°C for 15 s and 50°C for 30 s.

### Cytogenetic analysis of *Nosema* distribution in the ovaries of three *Muscidifurax* species

The ovaries of infected *M. zaraptor* and *M. raptor* were compared with the closely-related *Nosema*-free species *M. uniraptor*. Ovaries were fixed for 15 min in 10% Formaldehyde and 0.2% Tween in Phosphate Buffered Saline (PBS-T). Actin staining (Rhodamine Phalloidin, Invitrogen) was performed overnight or longer at 4°C in PBS-T. Ovaries were then washed 3X in PBS-T and moved to Vectashield with DAPI (Vector Labs) for at least 16 h before mounting. Microscopy was performed using a Leica SP5 point scanning confocal (63x/1.4 HCX PL Apo CS oil lens). Images were collected with LAS AF. Minor processing (Gaussian blur) was performed using FIJI.

## Data availability statement

The datasets presented in this study can be found in online repositories. The names of the repository/repositories and accession number(s) can be found at: https://www.ncbi.nlm.nih.gov/, JAIOKI000000000.

## Author contributions

XW and JW contributed to the conception and design of the study. XX and CG performed the insect rearing and sample collection. CG and RW performed the heat treatment experiments. XX performed the DNA and RNA sequencing experiments. XX and XW performed the genomic data analysis and wrote the first draft of the manuscript. DB performed cytogenetic experiments and analysis. XW, CG, DB, and JW provided samples, resources, and analysis tools. JW, CG, RW, and DB wrote sections of the manuscript. All authors contributed to the article and approved the submitted version.

## Funding

This project was supported by the Alabama Agricultural Experiment Station and the Hatch program (ALA05-2-18041) of the National Institute of Food and Agriculture, U.S. Department of Agriculture. XW was supported by the National Science Foundation award (1928770) and a laboratory start-up fund from Auburn University College Veterinary Medicine. XX was supported by the Auburn University Presidential Graduate Research Fellowship and the College of Veterinary Medicine Dean’s Fellowship. JW thanks for support from NSF 1950078.

## Conflict of interest

The authors declare that the research was conducted in the absence of any commercial or financial relationships that could be construed as a potential conflict of interest.

## Publisher’s note

All claims expressed in this article are solely those of the authors and do not necessarily represent those of their affiliated organizations, or those of the publisher, the editors and the reviewers. Any product that may be evaluated in this article, or claim that may be made by its manufacturer, is not guaranteed or endorsed by the publisher.

## Footnotes

^1^ http://www.repeatmasker.org
