# Supplementary material for: New insights into the genome and transmission of the microsporidian pathogen Nosema muscidifuracis
Source: Front Microbiol. 2023 Apr 13;14:1152586. doi: 10.3389/fmicb.2023.1152586 (PMC10133504; doi:10.3389/fmicb.2023.1152586)
Supplement: Supplementary file 1 [file Data_Sheet_1.PDF]

Table S1. Summary PacBio long-read and Illumina (10× Genomics) linked-read sequencing data generated for *Muscidifurax zaraptor* and *Nosema muscidifuracis* genome assembly.

Genome Statistics	*Muscidifurax zaraptor*	*Nosema muscidifuracis*
**Genome size (bp)**	386,836,632	14,397,169
**PacBio data: # of HiFi reads**	1,842,231	-
**PacBio: total HiFi sequences**	23.7 Gbp	-
**PacBio: average depth**	61.2×	258.2×
**Pacbio: % scaffold mapped**	99.98%	99.58%
**Illumina data: # of linked reads**	424,317,074	-
**Illumina: total sequences**	63.6 Gb	-
**Illumina: average depth**	54.5×	226.7×
**Illumina: % scaffold mapped**	99.95%	99.42%

Table S2. Average PacBio coverage depth against the *Nosema muscidifuracis* genome.

contig	length (bp)	covered %	depth
contig01	982164	99.99%	207.709
contig02	933025	100.00%	303.615
contig03	833206	99.76%	281.799
contig04	682066	100.00%	282.248
contig05	669200	98.96%	272.678
contig06	635296	99.83%	315.956
contig07	610585	98.52%	206.116
contig08	588184	99.98%	280.003
contig09	577561	99.78%	204.839
contig10	554543	100.00%	281.667
contig11	544348	99.75%	328.366
contig12	522755	99.70%	167.12
contig13	491432	98.60%	229.028
contig14	490971	100.00%	194.356
contig15	459695	99.79%	331.034
contig16	442036	99.98%	311.178
contig17	421492	99.76%	231.984
contig18	407860	99.99%	224.255
contig19	396865	99.67%	260.539
contig20	396051	98.77%	213.116
contig21	388771	100.00%	354.814
contig22	379414	100.00%	346.008
contig23	368489	100.00%	176.693
contig24	350964	99.41%	191.966
contig25	328750	99.80%	170.164
contig26	325008	98.41%	244.626
contig27	316965	100.00%	292.97
contig28	299473	100.00%	280.375

Table S3. Summary of annotated repeat elements in *Nosema muscidifuracis* genome.

Categories	# of elements	Length (% of genome)
**Retroelements**	1,008	863,391 (6%)
LINEs (RTE/Bov-B)	374	241,929 (1.68%)
LTR elements (Gypsy)	634	621,462 (4.32%)
**DNA transposons**	219	502,982 (3.49%)
**Unclassified**	1,462	2,396,435 (16.65%)
**Simple repeats**	3,313	246,854 (1.71%)
**Low complexity**	1,215	68,351 (0.47%)
**Total**	7,217	4,078,013 (28.33%)

Table S4. The annotation of noncoding RNAs in the *Nosema muscidifuracis* genome.

Type	Class	# of copy	Average length (bp)	Total length (bp)	% of genome
**tRNA**		170	75.1	12,765	0.089%
**rRNA**	5S	34	119.2	4,052	0.028%
	LSU	57	2315.1	131,958	0.917%
	SSU	57	1237.1	70,516	0.490%
	**Total rRNA**	**148**	**1395.4**	**206,526**	**1.434%**
**snRNA**	CD-box	2	179.0	358	0.002%
	splicing	7	142.6	998	0.007%
	**Total snRNA**	**9**	**150.7**	**1,356**	**0.009%**

Table S5. The 18S primers used for the quantification of *Nosema* titer in the *Muscidifurax zaraptor* genome.

Species name	Target gene	Primer name	Primer sequence	Product size	PCR Ta
Nosema muscidifuracis	18S	NP1_F	GAAGAAGTATCTGAAAAATGGAC	151 bp	49.3°C
Nosema muscidifuracis	18S	NP1_R	CGTTACTGCCTTGTTAAGCC		
Nosema muscidifuracis	18S	NP2_F	AAGAAGTATCTGAAAAATGG	213 bp	49.8°C
Nosema muscidifuracis	18S	NP2_R	CTTAGACTTAGTAGCCGTCTC		
Nosema muscidifuracis	18S	NP3_F	TTATAGACAGACACAATCAG	225 bp	49.7°C
Nosema muscidifuracis	18S	NP3_R	ATATCATCTTAGATAGCGACGG		

Table S6. RNA sequencing sample information, data yield, quality control summary statistics.

Library ID	Sex	Host	Replication information	# of paired-end reads	# of reads after QC	% of reads after QC
Mzar_adultF_FL_rep1	Female	fleshfly	replicate 1	85,853,492	84,584,114	98.52%
Mzar_adultF_FL_rep2	Female	fleshfly	replicate 2	57,015,645	56,227,715	98.62%
Mzar_adultF_FL_rep3	Female	fleshfly	replicate 3	69,336,408	68,326,961	98.54%
Mzar_adultF_HL_rep1	Female	house fly	replicate 1	76,735,261	75,692,065	98.64%
Mzar_adultF_HL_rep2	Female	house fly	replicate 2	77,203,402	75,967,748	98.40%
Mzar_adultF_HL_rep3	Female	house fly	replicate 3	56,990,139	56,109,147	98.45%
Mzar_adultM_FL_rep1	Male	fleshfly	replicate 1	49,640,375	48,903,854	98.52%
Mzar_adultM_FL_rep2	Male	fleshfly	replicate 2	60,168,883	59,363,068	98.66%
Mzar_adultM_FL_rep3	Male	fleshfly	replicate 3	61,031,813	60.048,859	98.39%
Mzar_adultM_HL_rep1	Male	house fly	replicate 1	57,421,992	56,504,800	98.40%
Mzar_adultM_HL_rep2	Male	house fly	replicate 2	51,761,573	50,944,361	98.42%
Mzar_adultM_HL_rep3	Male	house fly	replicate 3	64,986,450	64,014,005	98.50%
