# Supplementary material for: New insights into the genome and transmission of the microsporidian pathogen Nosema muscidifuracis
Source: Front Microbiol. 2023 Apr 13;14:1152586. doi: 10.3389/fmicb.2023.1152586 (PMC10133504; doi:10.3389/fmicb.2023.1152586)

Figure S1. The plot of GC content and CpG percentage versus average coverage for all scaffolds from the initial assembly suggested that the scaffolds of *N. muscidifuracis* are separated from other scaffolds in *M. zaraptor* genome.

(**A**) The plot showing the GC content versus average coverage for all scaffolds from the initial assembly. The scaffolds of *N. muscidifuracis* with higher coverage and extremely low GC content are plotted using blue dots, and the scaffolds in *M. zaraptor* genome are labeled in red. (**B**) The plot showing the CpG percentage and average coverage for all scaffolds from the initial assembly.

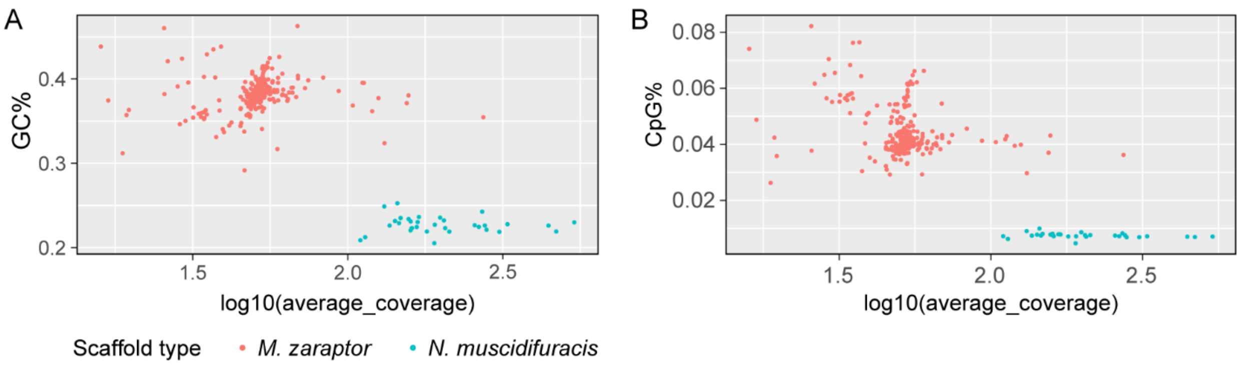

Figure S2. Genome comparisons between *Nosema muscidifuracis* and *Encephalitozoon cuniculi*.

A total of 24 contigs of *N. muscidifuracis* (86.6% of assembly in this research) show a one-to-one relationship with 11 scaffolds in the *E. cuniculi* genome (100% of assembly GCA_000091225.2). The contigs on the left of the circle represent *N. muscidifuracis* contigs, and the scaffolds on the right represent *E. cuniculi* scaffolds.

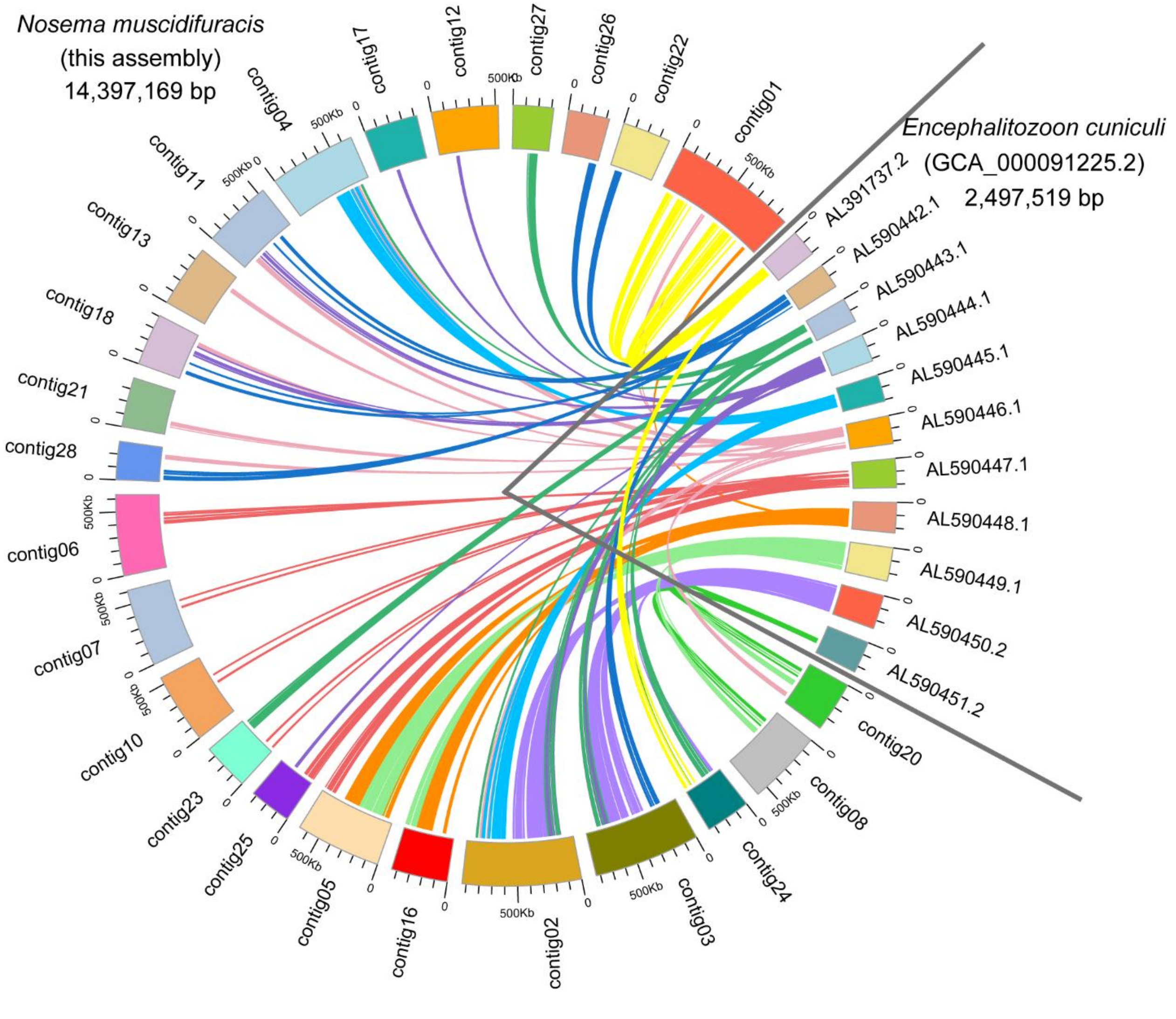

Figure S3. PCR results for three primer sets targeting the 18S rDNA gene in *Nosema muscidifuracis*.

The PCR amplification products for three 18S rDNA gene primer sets in *M. zaraptor* DNA sample were evaluated by a 2% agarose gel alongside a Quick-Load® 100 bp DNA Ladder, with *N. vit* (*Nasonia vitripennis*, *Nosema*-free) as a negative control. The gel was run at 120 V for 20 min. The sizes of PCR products for NP1, NP2, and NP3 are 151 bp, 213 bp, and 225 bp, respectively. NP1: 5'-GAAGAAGTATCTGAAAAATGGAC-3' and 5'-CGTTACTGCCTTGTTAAGCC-3'; NP2: 5'-AAGAAGTATCTGAAAAATGG-3' and 5'-CTTAGACTTAGTAGCCGTCTC-3'; NP3: 5'-TTATAGACAGACACAATCAG-3' and 5'-ATATCATCTTAGATAGCGACGG-3'.

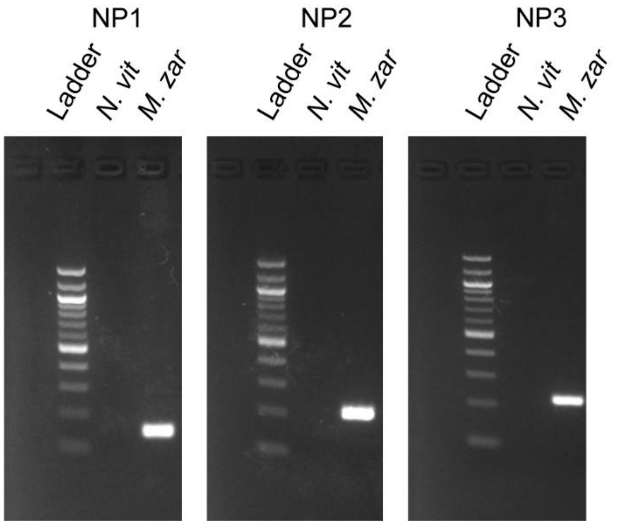

Supplement: Supplementary file 2 [file Data_Sheet_2.PDF]
